# “A Biomarker‐Based Scoring System to Assess the Presence of Obstructive Coronary Artery Disease in Patients With Myocardial Infarction”

**DOI:** 10.1002/clc.70090

**Published:** 2025-02-18

**Authors:** María Jesús Espinosa Pascual, Jose Antonio Carnicero Carreño, Mariam El Assar, Renee Olsen Rodríguez, Alfonso Fraile Sanz, Paula Rodriguez Montes, Nuria Gil Mancebo, Alberto Sánchez Ferrer, Bárbara Izquierdo Coronel, María Álvarez Bello, María Martín Muñoz, Verónica Cámara Hernández, Miguel de La Serna Real de Asua, Silvia Humanes Ybañez, Patricia Sosa Callejas, Miguel Gutierrez Muñoz, Rebeca Mata Caballero, Paula Awamleh Garcia, Jesús Ángel Perea Egido, Javier López Pais, Leocadio Rodríguez Mañas, Joaquín Jesús Alonso Martín

**Affiliations:** ^1^ Cardiology Department Hospital Universitario Getafe Getafe Spain; ^2^ Department of Medicine, Faculty of Biomedical and Health Sciences Universidad Europea de Madrid Villaviciosa de Odón Spain; ^3^ Aging and Frailty Department Fundación de Investigación Biomédica del Hospital Universitario de Getafe Getafe Spain; ^4^ Department of Clinical Analysis Hospital Universitario Getafe Getafe Spain; ^5^ Cardiology Department Hospital Clínico Universitario Santiago de Compostela, A Coruña Spain

**Keywords:** biomarkers, diagnosis, endothelial dysfunction, index, inflammation, MICAD, MINOCA, myocardial infarction, score

## Abstract

**Aims:**

Approximately 10% of patients with myocardial infarction present with non‐obstructive coronary arteries (MINOCA), whose characteristics differ from those with obstructive coronary lesions (MICAD). Inflammation plays a key role in myocardial infarction. This study aims to develop a biomarker‐based index for accurate differentiation between MINOCA and MICAD.

**Methods:**

A prospective, observational cohort study including 111 patients admitted for myocardial infarction: 46 with MINOCA and 65 with MICAD. Blood samples were collected within the first 24 h to measure high‐sensitivity C‐reactive protein, interleukin‐6, asymmetric dimethylarginine, and peak high‐sensitivity troponin T. The association of these biomarkers with MICAD risk was analyzed using logistic regression. Scoring systems were developed using optimization algorithms to predict the diagnosis before coronary angiography, applied to both individual biomarkers and a combined index.

**Results:**

Patients had a mean age of 67 years (SD 13.3), with a male predominance (68.5%). Higher levels of IL‐6 and high‐sensitivity troponin T were significantly associated with increased MICAD risk (OR: 1.58; 95% CI: 1.01–2.46, and OR: 2.27; 95% CI: 1.61–3.26, respectively). As score increases, interleukin‐6 and high‐sensitivity troponin T increase the likelihood of MICAD classification, while higher asymmetric dimethylarginine levels reduce it. Each one‐point increase in the combined index multiplies MICAD risk by six (OR:6.16, 95%CI: 2.72–13.95; *p* < 0.001). While individual indexes improved the diagnostic performance of biomarkers, the combined index demonstrated superior accuracy (AUC: 0.918).

**Conclusions:**

A biomarker‐based scoring system was developed, achieving superior discriminatory capacity for differentiating MINOCA from MICAD compared to the individual analysis of biomarkers in absolute values or independent indexes.

## Summary

- **What is known about the topic?**
  ◦ Patients with MINOCA have distinct characteristics compared to those with MICAD, with inflammation and endothelial dysfunction potentially serving as key differentiators.
  ◦ High‐sensitivity troponin, commonly used in clinical practice for diagnosing myocardial infarction, is valuable but does not allow for precise differentiation between MINOCA and MICAD when used alone.
  ◦ The integration of inflammatory and endothelial dysfunction biomarkers with troponin in a scoring system could potentially provide a more accurate method for distinguishing between these two types of infarction.
- **What's new?**
  ◦ This study is the first to develop a scoring system using circulating biomarkers to more accurately differenciate between MINOCA and MICAD.
  ◦ We focused on four key biomarkers: IL‐6, hs‐CRP, ADMA and hs‐troponin T. By combining troponin T, which is widely used in MI diagnosis, with inflammatory and endothelial dysfunction biomarkers, our approach captures the complex underlying processes involved in myocardial infarction.
  ◦ Our study found that combining these biomarkers into a single index demonstrated outstanding diagnostic discrimination, significantly improving the identification of MI type compared to using individual biomarkers alone.
  ◦ This innovative scoring system has the potential to optimize MI patient management and guide treatment strategies more accurately.

## Graphical abstract

Comparison of diagnostic performance in detecting obstructive coronary artery disease in patients with myocardial infarction: the biomarker‐based index demonstrates outstanding discrimination compared to individual biomarker levels (ln BM) and individual índices (4BM). AUC values for models based on log‐transformed biomarkers (ln BM), individual indices, and the combined four‐biomarker index (4BM) are shown. Abbreviations: AUC = area under the curve; MICAD = Myocardial Infarction with Obstructive Coronary Artery Disease; MINOCA = Myocardial Infarction with Non‐Obstructive Coronary Arteries; MI = myocardial infarction; IL‐6 = Interleukin‐6, hs‐CRP = high‐sensitivity C‐reactive protein, ADMA = Asymmetric Dimethylarginine, hs‐troponin T = High‐sensitivity troponin T.

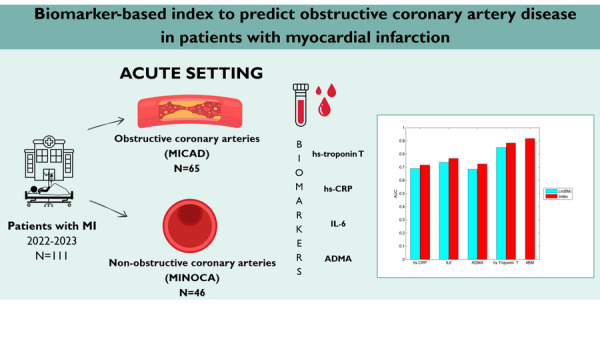

**Abbreviations**
ADMA: asymmetric dimethylarginine,
eGFR: glomerular filtration rate
hs‐CRP: high‐sensitivity c‐reactive protein
hs‐troponin T: high‐sensitivity troponin T
IL‐6: interleukin‐6
LVEF: left ventricular ejection fraction
MACE: major cardiovascular events
MI: myocardial infarction
MICAD: myocardial infarction with obstructive coronary artery disease
MINOCA: myocardial infarction with non‐obstructive coronary arteries
PCI: percutaneous coronary intervention
TIA: transient ischemic attack

## 1 Introduction

Approximately 10% of patients with myocardial infarction present with coronary arteries without significant stenosis (MINOCA) [1]. This group of patients exhibits distinct clinical and pathophysiological characteristics compared to those with myocardial infarction and obstructive coronary lesions (MICAD) [2]. However, the diagnosis and treatment of these patients represent a considerable challenge, requiring a personalized clinical approach to identify the underlying cause and establish an appropriate therapeutic strategy [3].

Systemic inflammation is recognized as a critical factor not only in the acute phase of myocardial infarction (MI) but also in driving the progression of atherosclerotic disease and plaque instability [4]. The growing importance of inflammatory biomarkers in cardiovascular research lies in their potential to enhance our understanding of underlying mechanisms and improve the identification, classification, and prognosis of affected patients [5]. Furthermore, inflammation is emerging as a promising therapeutic target, as anti‐inflammatory treatments have demonstrated the ability to reduce cardiovascular event rates in patients with myocardial infarction and chronic coronary artery disease [6, 7, 8, 9, 10]. Endothelial dysfunction plays an additional role in the pathophysiological processes of coronary artery disease [11].

Circulating biomarkers offer quantitative measures to assess key processes involved in MI, including cardiomyocites injury (high‐sensitivity troponin T), inflammation (high‐sensitivity CRP, IL‐6), and endothelial dysfunction (ADMA) [12]. Although several studies have shown that the inflammatory burden differs between patients with MINOCA and those with MICAD [13, 14, 15, 16, 17], suggesting that inflammatory biomarkers could potentially differentiate between these types of infarction before coronary angiography, this potential has not been fully explored. Such differentiation could enable earlier personalization of therapeutic strategies. Moreover, endothelial dysfunction in MINOCA remains under‐researched. Furthermore, no index combining multiple circulating biomarkers has been developed to diagnose the type of MI. Gaining further understanding of these biomarkers is essential for improving diagnosis accuracy and facilitating more personalized and targeted treatment approaches in MI patients.

The main objective of this study is to develop a biomarker‐based index that enables precise differentiation between myocardial infarction types: with and without significant obstructive coronary lesions, before coronary angiography.

## 2 Methods

### 2.1 Study Design and Population

A prospective, observational, and analytical cohort study was conducted. All patients admitted for MI to our hospital between 2022 and 2023 who underwent coronary angiography, agreed to participate in the study, and completed the biological sample collection protocol were included. Before inclusion, each participant signed an informed consent form. Based on angiographic findings, patients were classified into two groups: those with no significant coronary stenosis (MINOCA) and those with significant stenosis (MICAD). To minimize selection bias, for each MINOCA case, two MICAD cases were selected. However, parity was not fully achieved in the final sample due to some pairs lacking sufficient available biological samples to measure all the biomarkers included in the study, or, in certain instances, only one MICAD case could be identified that matched a given MINOCA case.

Patients diagnosed with Takotsubo syndrome or myocarditis were excluded from the analysis, in accordance with the definitions established by guidelines and scientific society positions. Additionally, patients with myocardial infarction and a positive SARS‐CoV‐2 test were excluded due to potential interference with the interpretation of the results, resulting in a final sample of 111 subjects. The study was approved by the hospital's ethics committee and followed national and international guidelines (Ethics Committee Code: 16/90). The study is registered in ClinicalTrials (ClinicalTrials.gov Identifier: NCT06446895).

### 2.2 Definitions

The diagnosis of MINOCA was based on the latest definitions from the ESC [3] and AHA [18]: (1) patients with MI according to the 4th universal definition [19]; (2) nonsignificant coronary lesions (stenosis < 50%); and (3) absence of an alternative cause for the MI. Patients with MI and significant stenosis (≥ 50%) were classified as MICAD.

### 2.3 Biomarker Analysis

Plasma and serum samples were collected via venipuncture within the first 24 h of symptom onset. These samples were stored in 0.3 mL aliquots at −80°C. IL‐6 and ADMA levels were measured in plasma using ELISA with commercial kits (Diaclone and bio‐Techne respectively) and analyzed using the FLUOstar Omega reader (BMG Labtech). Hs‐CRP and hs‐troponin T levels were determined in serum using particle‐enhanced immunoturbidimetry on a Cobas 8000 Roche analyzer.

### 2.4 Prognosis

Based on medical records and follow‐up calls, mortality from any cause and a composite of major adverse cardiovascular events (MACE) were determined. MACE was defined as cardiovascular death, readmission due to cardiovascular causes, reinfarction, and stroke. Cardiovascular readmissions included coronary causes, heart failure, or other cardiovascular‐related conditions. The median follow‐up duration was 13 months (interquartile range 6–23 months).

### 2.5 Indexes

Using numerical optimization algorithms, two scoring and stratification systems were designed: individual and combined, to maximize discrimination as measured by the AUC. In the individual system, five cut‐off points were estimated for each biomarker and event, generating a score ranging from 0 (lower probability of obstructive coronary artery disease in patients with myocardial infarction) to 5 (higher probability). In the combined system, five cut‐off points were estimated for each biomarker for each event, generating a score from 0 (lower probability) to 20 (higher probability).

### 2.6 Statistical Analysis

Descriptive values for sociodemographic and clinical variables are presented as means (SD) and frequencies (%), while biomarker values are presented as medians (IQR). Differences between groups were evaluated using the Mann–Whitney *U* test for continuous variables and the *χ* ^2^ test for categorical variables. Associations between biomarkers and the likelihood of being classified as MICAD were analyzed using a logistic regression model adjusted for age and sex through three approaches [1]: using each biomarker as a continuous variable [2], using each individual stratified index, and [3] using the combined index. An odds ratio (OR) < 1 indicates a higher likelihood of MINOCA, while an OR > 1 suggests a higher likelihood of MICAD. All analyses were performed using R for Windows v.4.1.2. Statistical significance was set at *p* < 0.05.

## 3 Results

### 3.1 Baseline Characteristics and Biomarker Levels

Among the 111 patients included, 46 were classified as MINOCA and 65 as MICAD. The mean age was 67 years (SD 13.3), and 68.5% were male. Fifteen patients died during the study follow‐up. Personal medical history, baseline characteristics at admission and during hospitalization, along with data on mortality and MACE are detailed in Table 1.

**Table 1:** Personal history, clinical characteristics at admission, during hospitalization, and follow‐up of patients with acute myocardial infarction, according to the presence or absence of obstructive coronary artery disease.

	TOTAL	MICAD	MINOCA	*p*‐value
	(*N* = 111)	(*N* = 65)	(*N* = 46)	
Risk factors				
Age (years), median (IQR)	67.0 (13.3)	68.7 (13.5)	64.6 (12.7)	0.113
Male gender, *n* (%)	76 (68.5)	52 (80.0)	24 (52.2)	**0.002**
Diabetes, *n* (%)	33 (29.7)	23 (35.4)	10 (21.7)	0.121
Dyslipidemia, *n* (%)	57 (51.4)	38 (58.5)	19 (41.3)	0.075
Hypertension, *n* (%)	70 (63.1)	40 (61.5)	30 (65.2)	0.692
*Tobacco consumption, *n* (%)	75 (67.6)	46 (70.8)	28 (63.0)	0.392
Personal history				
Previous MI, *n* (%)	20 (18.0)	14 (21.5)	6 (13.0)	0.251
Previous PCI, *n* (%)	20 (18.0)	14 (21.5)	6 (13.0)	0.251
Chronic heart failure, *n* (%)	8 (7.2)	6 (9.2)	2 (4.3)	0.327
Previous TIA or stroke, *n* (%)	14 (12.6)	10 (15.4)	4 (8.7)	0.296
**Chronic Kidney Disease, *n* (%)	22 (19.8)	16 (24.6)	6 (13.0)	0.132
***Chronic Respiratory Disease, *n* (%)	15 (13.5)	9 (13.9)	6 (13.0)	0.903
Atrial fibrillation/flutter, *n* (%)	18 (16.4)	13 (20.0)	5 (11.1)	0.215
Gastroesophageal Disease, *n* (%)	21 (18.9)	6 (9.2)	15 (32.6)	**0.002**
No emotional stress, *n* (%)	42 (37.8)	30 (46.1)	12 (26.1)	**0.032**
Treatment at admission				
Aspirin	25 (22.5)	16 (24.6)	9 (19.6)	0.530
Anticoagulants	19 (17.1)	12 (19.5)	7 (16.2)	0.655
Vitamin K antagonists	8 (7.2)	4 (6.2)	4 (8.7)	
Apixaban	5 (4.5)	4 (6.2)	1 (2.2)	
Rivaroxaban	1 (0.9)	0 (0.0)	1 (2.2)	
Edoxaban	5 (4.5)	4 (6.2)	1 (2.2)	
Dabigatran	0 (0.0)	0 (0.0)	0 (0.0)	
Beta‐blockers	30 (27.0)	18 (27.7)	12 (26.1)	0.851
ACE inhibitors	36 (32.4)	16 (24.6)	20 (43.5)	**0.037**
Angiotensin II receptor antagonists	19 (17.1)	13 (20.0)	6 (13.0)	0.338
Nitrates	11 (9.9)	6 (9.2)	5 (10.9)	0.776
Calcium antagonists	19 (17.1)	13 (20.0)	6 (13.0)	0.338
Statins	49 (44.5)	32 (49.2)	17 (37.8)	0.235
Clinical, laboratory and ECG findings on admission				
Heart Rate (bpm), median (IQR)	82.4 (20.7)	80.2 (20.1)	85.6 (21.4)	0.147
Systolic Blood Pressure (mmHg), median (IQR)	144.6 (31.6)	143.2 (25.1)	146.6 (39.1)	0.895
Repolarization, *n* (%)				**0.001**
Normal, *n* (%)	27 (24.3)	8 (12.3)	19 (41.3)	
ST elevated, *n* (%)	23 (20.7)	20 (30.8)	3 (6.5)	
ST depressed, *n* (%)	28 (25.2)	17 (26.1)	11 (23.9)	
T negative, *n* (%)	20 (18.0)	12 (18.5)	8 (17.4)	
Hemoglobin (g/dL), mean (SE)	13.7 (2.3)	13.6 (2.3)	13.9 (2.3)	0.466
Creatinine (mg/dL), mean (SE)	1.2 (0.8)	1.2 (0.7)	1.1 (0.8)	0.100
Cholesterol (mg/dL), mean (SE)	168.6 (50.8)	161.0 (48.7)	179.3 (52.2)	0.080
During hospitalization				
Normal LVEF, *n* (%)	58 (52.3)	27 (41.5)	31 (67.4)	**0.007**
Follow‐up				
MACE, *n* (%)	27 (25.0)	21 (33.9)	6 (13.0)	**0.013**
Cardiovascular mortality, *n* (%)	7 (46.7)	7 (46.7)	0 (NaN)	NaN
Cardiovascular readmission, *n* (%)	19 (17.8)	14 (22.9)	5 (10.9)	0.106
TIA or stroke, *n* (%)	5 (4.7)	2 (3.3)	3 (6.5)	0.431
Recurrent myocardial infarction	6 (5.6)	5 (8.2)	1 (2.2)	0.180

*Note:* Continuous variables presented as mean ± SD. Categorical variables presented as *N* (%). Bolded values are statistically significant (*p* < 0.05). *Tobacco consumption is defined as active and ex‐smokers. **Chronic Kidney Disease is defined as eGFR < 60). GFR = Glomerular Filtration Rate, ***Chronic Respiratory Disease is defined as Chronic Obstructive Pulmonary Disease, asthma, or Sleep Apnea.

Abbreviations: LVEF = left ventricular ejection fraction, MACE = major adverse cardiac events, MI = myocardial infarction, MICAD = myocardial infarction with obstructive coronary artery disease, MINOCA = myocardial infarction with non‐obstructive coronary arteries, NA = Not applicable, PCI = percutaneous coronary intervention, TIA = transient ischemic attack.

In patients with MINOCA, a higher proportion of women was observed (47.8% vs. 20%, *p* = 0.0019) compared to those with MICAD. MINOCA patients also had a higher prevalence of gastroesophageal disease (32.6% vs. 9.2%, *p* = 0.002) and self‐reported stress (73.9% vs. 53.9%, *p* = 0.032). The underlying mechanisms of MINOCA, included: unknown (49.1%), Type II (30.2%), plaque rupture with probably thrombus lysis at the time of catheterization (9.3%), vasospasm (4.5%), microvascular injury (2.3%), coronary dissection (2.3%), and Kounis syndrome (2.3%). MINOCA patients had significantly lower peak levels of hs‐troponin T compared to those with MICAD (259.1 ng/L vs. 2032.3 ng/L, *p* < 0.001), and the percentage of patients with a normal left ventricular ejection fraction (LVEF > 55%) was significantly higher (67.4% vs. 41.5%, *p* = 0.007). The MICAD group had a higher MACE during follow‐up (*p* = 0.0134).

During the acute phase, IL‐6 levels (7.4 vs 4.1 mg/dL; *p* = 0.005) were significantly higher in MICAD patients. Table 2 shows the baseline levels of the four biomarkers analyzed. Figure S1 presents violin plots of the log‐transformed (ln) biomarker levels in relation to the presence of obstructive coronary artery disease in patients with myocardial infarction.

**Table 2:** Biomarker levels at admission in patients with acute myocardial infarction, according to the presence or absence of obstructive coronary artery disease.

Inflammatory biomarkers Median (IQR)	Total	MICAD	MINOCA	*p*‐value
	(*N* = 111)	(*N* = 65)	(*N* = 46)	
hs‐CRP (mg/dL)	3.2 (0.9, 9.3)	3.2 (1.2, 9.5)	3.1 (0.8, 9.2)	0.522
IL‐6 (pg/mL)	5.5 (3.0, 12.8)	7.4 (4.1, 15.5)	4.1 (2.3, 8.3)	**0.005**
ADMA (ng/mL)	237.9 (182.4, 392.1)	235.0 (198.9, 331.8)	255.4 (173.4, 412.8)	0.865
hs‐troponin T (ng/mL)	311.0 (83.0, 1286.8)	858.0 (195.8, 2756.5)	95.2 (46.0, 228.0)	**< 0.001**

*Note:* Median values with interquartile ranges (IQR) are presented. Bolded values are statistically significant (*p* < 0.05).

Abbreviations: ADMA = asymmetric dimethylarginine, hs‐CRP = high‐sensitivity C‐reactive protein, hs‐troponin T = high‐sensitivity troponin T, IL‐6 = interleukin‐6, MICAD = myocardial infarction with obstructive coronary artery disease, MINOCA = myocardial infarction with non‐obstructive coronary arteries.

### 3.2 Associations Between Biomarkers and MICAD Risk

Logistic regression models adjusted for age and sex (Table 3) identified a significant association between admission levels of IL‐6 (OR: 1.58, 95% CI: 1.01–2.46; *p* = 0.045) and high‐sensitivity troponin T (OR: 2.27, 95% CI: 1.61–3.21; *p* < 0.001) with an increased risk of MICAD.

**Table 3:** Associations of biomarker levels with the risk of obstructive coronary artery disease in patients with myocardial infarction: logistic regression adjusted for age and sex, AUC, and *p*‐values.

**Levels of biomarkers (ln)**	**MICAD**			
	**OR (CI 95%)**	***p*‐value**	**AUC**	***p*‐value in relation to the combined index**
hs‐CRP (mg/dL)	1.03 (0.79, 1.35)	0.814	0.690	**< 0.001**
IL‐6 (pg/mL)	1.58 (1.01, 2.46)	**0.045**	0.736	**< 0.001**
ADMA (ng/mL)	0.87 (0.40, 1.90)	0.730	0.685	**< 0.001**
hs‐troponin T (ng/mL)	2.27 (1.61, 3.21)	**< 0.001**	0.850	**0.022**

*Note:* Odds ratios are presented for the association between log‐transformed biomarker levels and mortality. Bolded values are statistically significant (*p* < 0.05).

Abbreviations: ADMA = asymmetric dimethylarginine, AUC = area under the curve, hs‐CRP = high‐sensitivity C‐reactive protein, hs‐troponin T = high‐sensitivity troponin T, IL‐6 = interleukin‐6; MICAD = myocardial infarction with obstructive coronary artery disease, OR = odds ratios.

### 3.3 Development of a Biomarker‐Based Index

Using a numerical optimization algorithm, we developed the scoring system described in Supporting Information S1: Table S1. This model demonstrated that each one‐point increase in the index multiplies the risk of MICAD by six (OR: 6.16, 95% CI: 2.72–13.95; *p* < 0.001). The index exhibited an outstanding discriminative ability, with an AUC of 0.918.

Using the developed model, MICAD probabilities were calculated based on index scores, stratified by age and sex. Supporting Information S1: Table S2 presents the estimated probabilities of MICAD according to index score, age, and sex for men and women aged 50, 65, and 80 years. Supporting Information S1: Figure S2 offers a graphical representation of these probabilities to facilitate interpretation. Women with MI exhibited a significantly lower risk of MICAD compared to men (OR: 4.93, 95% CI: 1.48–16.37; *p* = 0.009). This can be seen in the continuous lines of the graph, which represent women and are shifted to the right, indicating that women require a higher index score to reach the same risk level as men. For instance, a 50‐year‐old man has a higher risk than an 80‐year‐old woman when both have the same index score.

Additionally, an analysis was conducted to determine if there was any point that could improve the predictive capacity of the score. It was identified that a score ≥ 6 provides a predictive capacity with an AUC of 0.867, which is lower than the AUC obtained when the score is used as a continuous variable.

**Figure 1:**
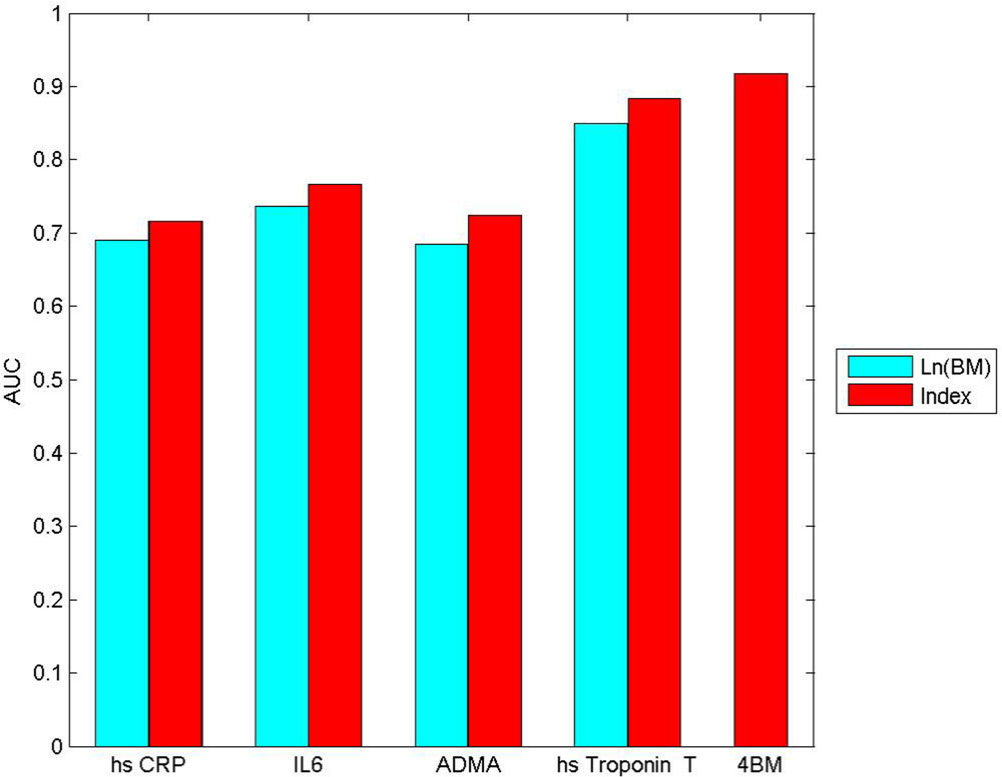
Representation of the areas under the curve (AUC) of the different models based on the ln‐transformed biomarkers (ln BM), individual indices, and the combined index (4BM). IL‐6 = Interleukin‐6, hs‐CRP = high‐sensitivity C‐reactive protein, ADMA = Asymmetric Dimethylarginine, hs‐troponin T = High‐sensitivity Troponin T.

Additionally, the predictive capacity of each biomarker was evaluated individually by creating specific scores for each one. This enabled the analysis of how effective each biomarker is on its own in distinguishing between the types of myocardial infarction. Table 4 shows the ORs of the indices and the corresponding AUC for each biomarker and the combined index. The results indicate that as the score increases, both IL‐6 and troponin Ths raise the risk of being classified as MICAD, while higher levels of ADMA reduce this probability by 29%. See Supporting Information S1: Figure S3. Figure 1 shows the AUC for the models developed (ln‐transformed biomarkers, individual indices, and the combined index). Table S3 in the supporting Information presents the scoring system developed for each biomarker, designed to categorize the type of myocardial infarction as either MINOCA or MICAD.

**Table 4:** Indexes of each individual biomarker and the combined index of the four biomarkers, in relation to the presence of obstructive coronary artery disease in patients with myocardial infarction. Odds ratios, *p*‐values and areas under the curve for individual and combined indices.

Index of biomarkers	MICAD			
	OR (CI 95%)	*p*‐value	AUC	*p*‐value in relation to the combined index
hs‐CRP	1.57 (0.87, 2.84)	0.133	0.716	**< 0.001**
IL‐6	1.79 (1.16, 2.76)	**0.008**	0.767	**< 0.001**
ADMA	0.71 (0.52, 0.97)	**0.030**	0.724	**< 0.001**
hs‐troponin T	3.65 (2.06, 6.44)	**< 0.001**	0.883	0.239
Combined index	6,16 (2.72, 13.95)	**< 0.001**	0.918	

*Note:* Odds ratios are presented for the association between log‐transformed biomarker levels and mortality. Bolded values are statistically significant (*p* < 0.05).

Abbreviations: ADMA = asymmetric dimethylarginine, AUC = area under the curve, hs‐CRP = high‐sensitivity C‐reactive protein, hs‐troponin T = high‐sensitivity troponin T, IL‐6 = interleukin‐6, MICAD = myocardial infarction with obstructive coronary artery disease, OR = odds ratios.

## 4 Discussion

This study presents the development of the first scoring system based on biomarkers to differentiate between MINOCA and MICAD. Although high‐sensitivity troponin is routinely used in the management of all MI patients and its clinical utility is well established [3], our results demonstrate a significant improvement when using its individual index to discriminate between MI types. Moreover, its diagnostic accuracy is further enhanced when complemented with inflammatory and endothelial dysfunction biomarkers. This integration allows for more precise classification and better differentiation between this two types of MI.

Although the levels of some biomarkers were significantly associated with MICAD, the creation of specific indices for each biomarker allowed for the identification of other associations, such as the inverse relationship with ADMA levels, thereby increasing the diagnostic accuracy. This is because the behavior of many biological variables does not follow a simple level‐dependent relationship [20]. These indices provide clear and easily applicable cut‐off points, making them a practical tool for daily clinical practice, as they could be automatically implemented in clinical laboratory computer systems, facilitating the identification of MI subtypes and optimizing clinical management.

Notably, the combined index of the four biomarkers showed exceptional diagnostic discrimination, offering a more robust diagnostic tool for MI type than the individual values of each biomarker. It accentuates the value of a comprehensive profile that integrates both inflammatory and endothelial dysfunction biomarkers, supporting the current perspective that the combination of circulating biomarkers provides a more accurate reflection of the complex pathological processes underlying MI. The role of hs‐CRP in enhancing the performance of the combined index, however, remains controversial.

Scoring systems have gained recognition for risk stratification in the cardiovascular field, particularly in the context of coronary atherosclerosis and acute coronary syndrome [21, 22, 23, 24]. However, traditional methods often rely on clinical parameters, acute‐phase reactants, or immune responses, without incorporating specific biomarkers that reflect the underlying pathological mechanisms. Our scoring system is distinctive in that it focuses on specific biomarkers related to inflammation and endothelial dysfunction, processes that appear to differ between patients with MINOCA and MICAD. These differences highlight the distinct pathological mechanisms underlying each condition, which may contribute to variations in the clinical characteristics and outcomes of patients. A key strength of this system is its simplicity and practical applicability, making it well‐suited for routine clinical practice to differentiate between these two types of MI.

Despite extensive research on inflammatory biomarkers in MI, few studies have compared these biomarkers in MINOCA and MICAD. The results have been inconsistent. Some groups have found higher hs‐CRP levels in MINOCA patients [15, 17], while our results align with Kallmeyer et al., who observed higher hs‐CRP in MICAD patients [16]. The inconsistency in results may be attributed to variations in adherence to current MINOCA definitions and differing inclusion criteria across studies. However, a consistent finding is the lower hs‐troponin T levels in MINOCA.

### 4.1 Limitations

One limitation of this study is the potential variability in biomarker measurements due to differences in commercial kits and the use of plasma or serum as the analytical matrix. This means that the absolute values of the biomarkers could vary between studies conducted at other centers. As a result, absolute biomarker values may differ across studies conducted at different centers. However, applying the same optimization methodology or using correction factors provided by manufacturers allows for the adjustment of cut‐off points, ensuring consistent results across various clinical settings. This adaptability increases the potential for applying the developed index to diverse populations and clinical contexts. Finally, although the combined‐index demonstrated exceptional performance and outperformed the analysis of individual biomarkers, it is essential to validate its applicability and robustness in larger, more diverse cohorts. Such validation would serve to reinforce the promising findings from this exploratory study, ensuring its broader clinical applicability and reliability across diverse patient populations and healthcare environments.

### 4.2 Clinical Relevance

This scoring system, which integrates circulating biomarkers indicative of myocardial damage, inflammation, and endothelial dysfunction, presents an innovative approach with significant potential for differenciate between obstructive and non‐obstructive coronary disease. Designed for quick, reliable interpretation, this index is a key tool for accurate MI classification and personalized management. By providing a deeper understanding of the mechanisms behind these two MI types, our system aims to improve diagnostic accuracy, guide targeted therapies, and ultimately improve patient outcomes.

While the scoring system proposed in this study may help prioritize patients for diagnostic procedures, it should not replace coronary angiography, which remains the gold standard for assessing coronary artery disease in MI patients. The scoring system can complement existing diagnostic tools by providing additional information on the patient's MI profile, thereby supporting clinical decision‐making and improving resource allocation.

## 5 Conclusion

Patients with MICAD had higher levels of IL‐6 and hs‐Troponin T compared to those with MINOCA. The combined biomarker index demonstrated outstanding diagnostic accuracy in identifying obstructive coronary artery disease in patients with MI, outperforming the use of individual biomarkers alone.

## Author Contributions

M.J.E.P.: conceptualization, design of the study, methodology, patient inclusión, data interpretation, writing–original draft, review and editing. J.C.C.: statistical analyses, methodology, data interpretation. M.E.A., V.C., and P.S.C.: biomarker analysis. A.S.F.: sample processing. M.M.M., M.A.B., B.I.C., J.L.P., C.P.A., R.O.R., A.F.S., S.H.Y., M.D.R., R.M.C., N.G.M., P.R.M.: patient inclusion in the database. J.P.E.: critical review. L.R.M.: provision of resources, supervisión. J.J.A.M.: conceptualization, critical review, supervision. All authors read and approved the final manuscript.

## Conflicts of Interest

The authors declare no conflicts of interest.

## Supporting information

Supporting information.

## Data Availability

The data that support the findings of this study are available on request from the corresponding author. The data are not publicly available due to privacy or ethical restrictions.
