# Supplementary material for: “A Biomarker‐Based Scoring System to Assess the Presence of Obstructive Coronary Artery Disease in Patients With Myocardial Infarction”
Source: Clin Cardiol. 2025 Feb 18;48(2):e70090. doi: 10.1002/clc.70090 (PMC11836528; doi:10.1002/clc.70090)

**SUPPLEMENTAL TABLES**

**Table S1.** Scores of individual biomarker scoring systems to determine the presence of obstructive coronary artery disease in patients with myocardial infarction

| **Biomarkers** | **Value range** | **Score** |  |
| --- | --- | --- | --- |
|  |  |  |  |
| hs-CRP (mg/dl) | 0 < x ≤ 48.93 | 0 |  |
|  | 48.93 < x ≤ 50.08 | 1 |  |
|  | 50.08 < x ≤ 274.02 | 2 |  |
|  | 274.02 < x ≤ 290.29 | 3 |  |
|  | 290.29 < x ≤ 313.67 | 4 |  |
|  | x > 313.67 | 5 |  |
| IL-6 (pg/dl) | 0 < x ≤ 0.65 | 0 |  |
|  | 0.65 < x ≤ 2.31 | 1 |  |
|  | 2.31 < x ≤ 41.07 | 2 |  |
|  | 41.07 < x ≤ 43.75 | 3 |  |
|  | 43.75 < x ≤ 87.51 | 4 |  |
|  | x > 87.51 | 5 |  |
| ADMA (ng/ml) | 0 < x ≤ 104.7 | 0 |  |
|  | 104.7 < x ≤ 111.18 | 1 |  |
|  | 111.18 < x ≤ 112.24 | 2 |  |
|  | 112.24 < x ≤ 1027.5 | 3 |  |
|  | 1027.5 < x ≤ 1038.51 | 4 |  |
|  | x > 1038.51 | 5 |  |
| hs-troponin T (ng/ml) | 0 < x ≤ 115.74 | 0 |  |
|  | 115.74 < x ≤ 1330.58 | 1 |  |
|  | 1330.58 < x ≤ 248.62 | 2 |  |
|  | 2485.62 < x ≤ 2509.1 | 3 |  |
|  | 2509.1 < x ≤ 16740.62 | 4 |  |
|  | x > 16740.62 | 5 |  |

*Notes:* Each biomarker score ranges from 0 to 5, where 0 indicates the lowest likelihood and 5 represents the highest likelihood of obstructive coronary artery disease in patients with myocardial infarction. Abbreviations: IL-6 = interleukin-6, hs-CRP = high-sensitivity C-reactive protein, ADMA = Asymmetric Dimethylarginine, hs-troponin T = high-sensitivity troponin T.

**Tabla S2. Probabilities of obstructive coronary artery disease according to index score, age, and sex for men and women aged 50, 65, and 80 years.**

| **Index score** | **Estimated Probabilities of MICAD** | | | | | |  |
| --- | --- | --- | --- | --- | --- | --- | --- |
|  |  |  |  |  |  |  |  |
|  | **Woman-50 y.o.** | **Man-50 y.o.** | **Woman-65 y.o.** | **Man-65 y.o.** | **Woman-80 y.o.** | **Man-80 y.o.** |  |
| 0 | 0 | 0 | 0 | 0 | 0 | 0 |  |
| 1 | 0 | 0 | 0 | 0 | 0 | 0.001 |  |
| 2 | 0 | 0.001 | 0.001 | 0.002 | 0.001 | 0.005 |  |
| 3 | 0.002 | 0.008 | 0.003 | 0.015 | 0.006 | 0.030 |  |
| 4 | 0.009 | 0.045 | 0.019 | 0.087 | 0.038 | 0.162 |  |
| 5 | 0.055 | 0.224 | 0.106 | 0.369 | 0.195 | 0.543 |  |
| 6 | 0.265 | 0.640 | 0.423 | 0.783 | 0.598 | 0.880 |  |
| 7 | 0.690 | 0.916 | 0.819 | 0.957 | 0.902 | 0.978 |  |
| 8 | 0.932 | 0.985 | 0.965 | 0.993 | 0.983 | 0.996 |  |
| 9 | 0.988 | 0.998 | 0.994 | 0.999 | 0.997 | 0.999 |  |
| 10 | 0.998 | 1.000 | 0.999 | 1 | 1 | 1 |  |
| > 11 | 1 | 1 | 1 | 1 | 1 | 1 |  |

*Notes:* The probabilities are based on the biomarker scoring system, where the index score ranges from 0 to 20, with higher scores indicating a greater likelihood of obstructive coronary artery disease. Abbreviations: MICAD = Myocardial Infarction with Obstructive Coronary Artery Disease, y.o. = years old.

**Tabla S3. Scoring System for the combined index to determine the presence of obstructive coronary artery disease in patients with myocardial infarction.**

| **Biomarkers** | **Value range** | **Score** |  |
| --- | --- | --- | --- |
|  |  |  |  |
| hs-CRP (mg/dl) | 0 < x ≤ 1.34 | 0 |  |
|  | 1.34 < x ≤ 46.81 | 1 |  |
|  | 46.81 < x ≤ 46.97 | 2 |  |
|  | 46.97 < x ≤ 256.08 | 3 |  |
|  | 256.08 < x ≤ 280.81 | 4 |  |
|  | x > 280.81 | 5 |  |
| IL-6 (pg/dl) | 0 < x ≤ 0.76 | 0 |  |
|  | 0.76 < x ≤ 4.17 | 1 |  |
|  | 4.17 < x ≤ 13.15 | 2 |  |
|  | 13.15 < x ≤ 13.26 | 3 |  |
|  | 13.26 < x ≤ 66.57 | 4 |  |
|  | x > 66.57 | 5 |  |
| ADMA (ng/ml) | 0 < x ≤ 309.8 | 0 |  |
|  | 309.8 < x ≤ 391.81 | 1 |  |
|  | 391.81 < x ≤ 408.05 | 2 |  |
|  | 408.05 < x ≤ 712.12 | 3 |  |
|  | 712.12 < x ≤ 717.31 | 4 |  |
|  | x > 717.31 | 5 |  |
| hs-troponin T (ng/ml) | 0 < x ≤ 116.01 | 0 |  |
|  | 116.01 < x ≤ 440.07 | 1 |  |
|  | 440.07 < x ≤ 1313.96 | 2 |  |
|  | 1313.96 < x ≤ 2258.78 | 3 |  |
|  | 2258.78 < x ≤ 2608.27 | 4 |  |
|  | x > 2608.27 | 5 |  |

*Notes:* The combined biomarker score ranges from 0 to 20, where 0 indicates the lowest likelihood of obstructive coronary artery disease and 20 represents the highest likelihood of obstructive coronary artery disease in patients with myocardial infarction. Abbreviations: IL-6 = interleukin-6, hs-CRP = high-sensitivity C-reactive protein, ADMA = Asymmetric Dimethylarginine, hs-troponin T = high-sensitivity troponin T.

**SUPPLEMENTAL FIGURES**

**Figure S1.** Violin plots of the log-transformed (ln) levels of biomarkers in relation to the presence of obstructive coronary artery disease in patients with myocardial infarction. Blue represents myocardial infarction with non-obstructive coronary arteries and red represents myocardial infarction with obstructive coronary artery disease. Abbreviations: MINOCA = Myocardial Infarction with Non-Obstructive Coronary Arteries, MICAD = Myocardial Infarction with Obstructive Coronary Artery DiseaseIL-6 = interleukin-6, hs-CRP = high-sensitivity C-reactive protein, ADMA = Asymmetric Dimethylarginine, hs-troponin T = high-sensitivity troponin T.


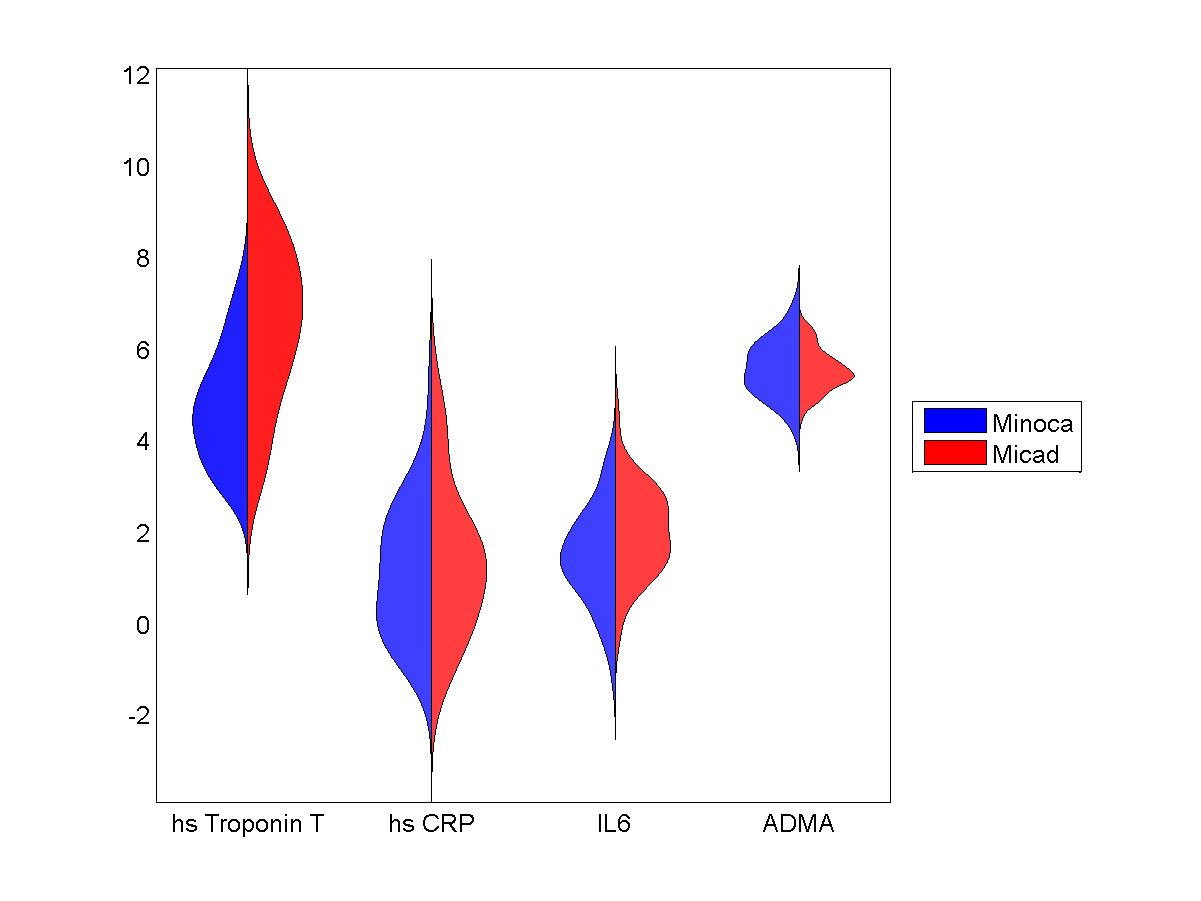


**Figure S2**: Probability of MICAD based on score values across different ages and sexes. Abbreviations: W-50 = Woman 50 years, M-50 = Man 50 years, W-65 = Woman 65 years, M-65 = Man 65 years, W-80 = Woman 80 years, M-80 = Man 80 years.


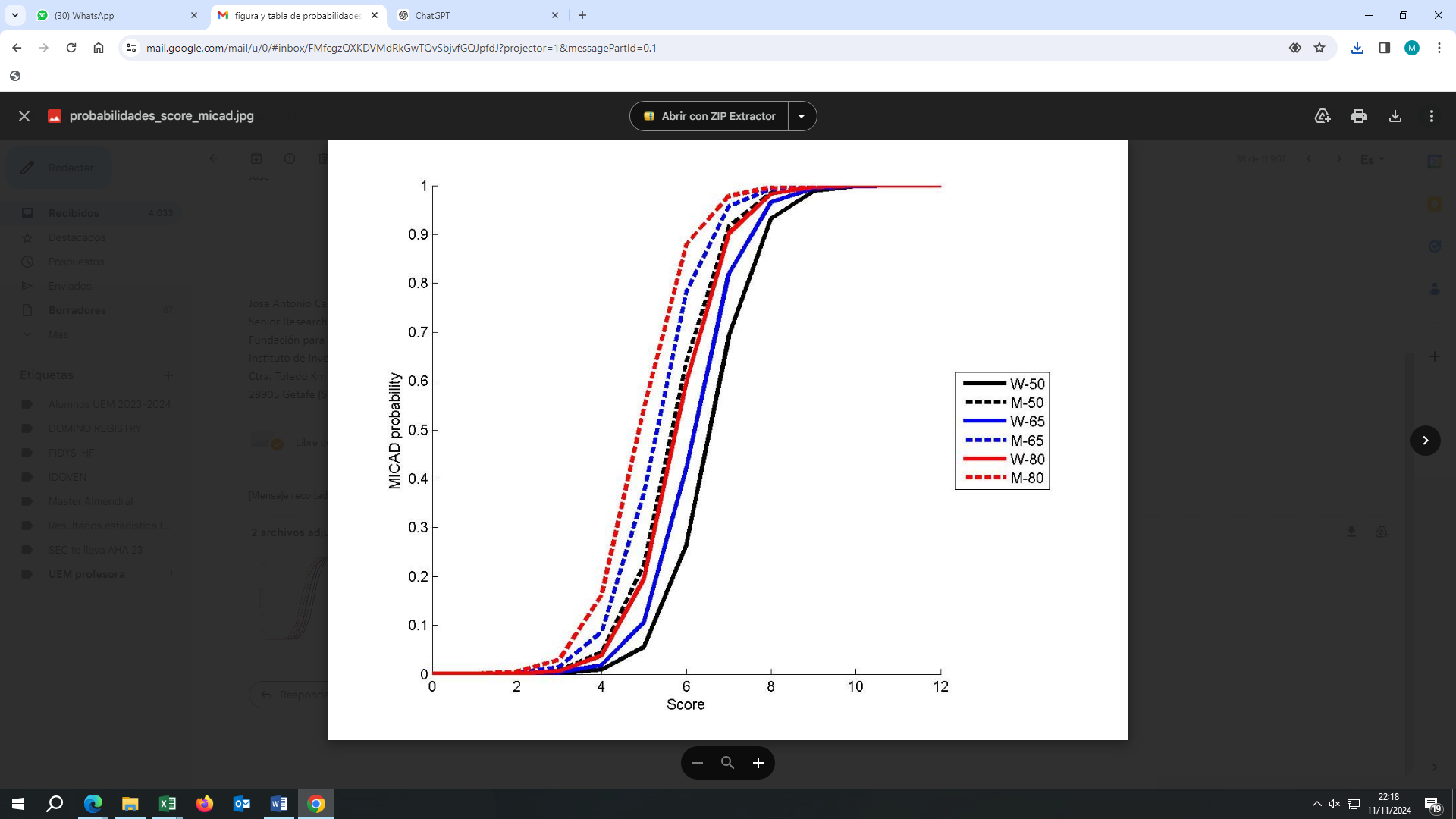


**Figure S3**: Representation of the odds ratios from logistic regression models including continuous biomarkers (c), individual indices (i), and the combined index (4BM), in relation to obstructive coronary artery disease in patients with myocardial infarction. The OR values are presented along with their confidence intervals. Abbreviations: IL-6 = Interleukin-6, hs-CRP = high-sensitivity C-reactive protein, ADMA = Asymmetric Dimethylarginine, hs-troponin T = High-sensitivity Troponin T.


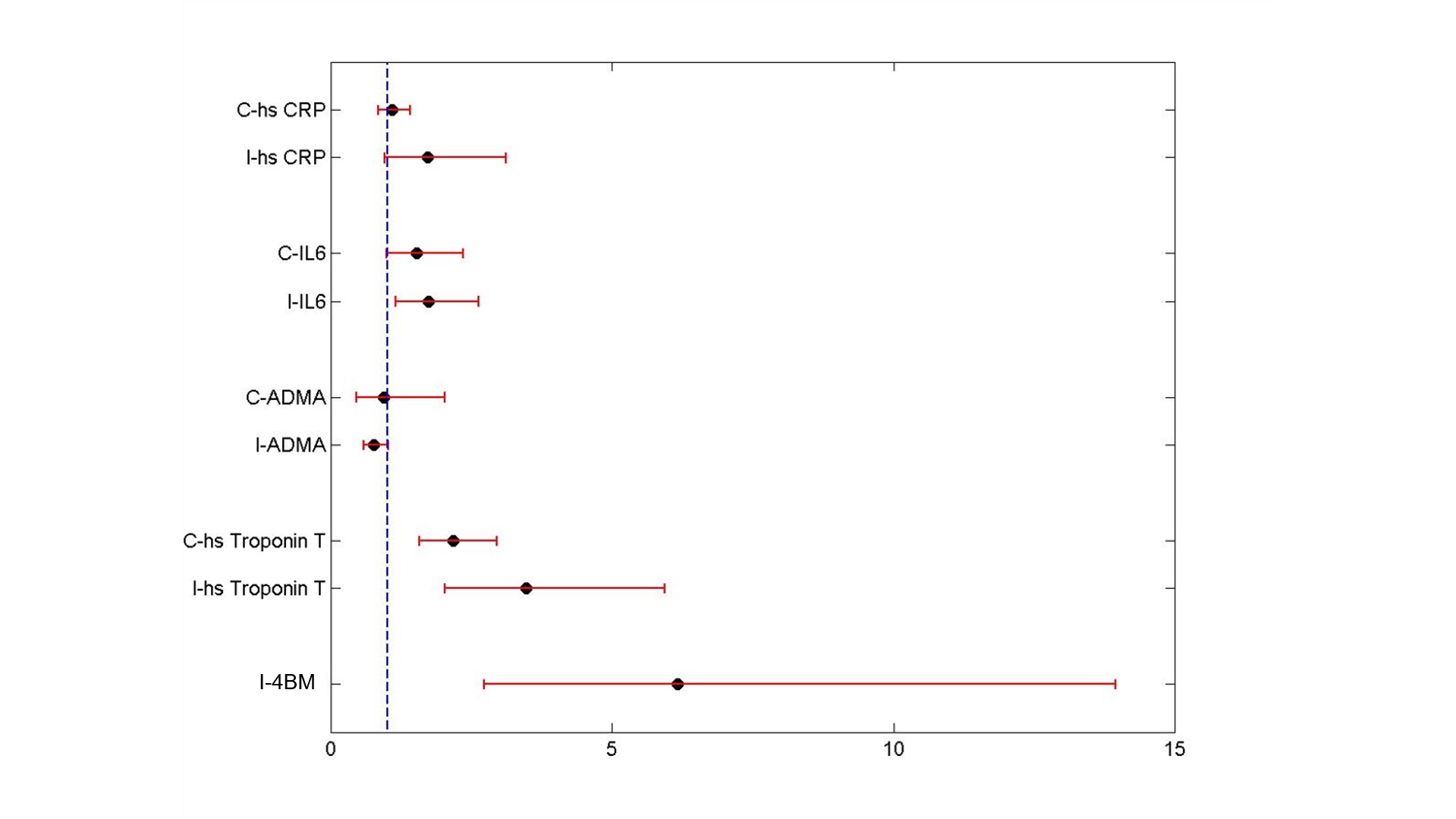

Supplement: Supplementary file 1 — Supporting information. [file CLC-48-e70090-s001.doc]
